# Green tea containing inulin and catechins ameliorates high-fat diet-induced metabolic dysfunction-associated fatty liver disease in mice

**DOI:** 10.7150/ijms.122441

**Published:** 2025-10-20

**Authors:** Yu-Hao Lee, Chi-Chang Huang, Hung-Lung Lin, Yi-Xuan Chen, Yi-Ju Hsu

**Affiliations:** 1 Graduate Institute of Sports Science, National Taiwan Sport University, Taoyuan City 333325, Taiwan.; 2 Department of Physical Medicine and Rehabilitation, Taipei Medical University-Hsin Kuo Min Hospital, Taoyuan City 333325, Taiwan.; 3 Department of Physical Medicine and Rehabilitation, School of Medicine, College of Medicine, Taipei Medical University, Taipei 11031, Taiwan.; 4 Research and Development, Vitalon Foods Co., Ltd, Taichung 407, Taiwan.

**Keywords:** inulin, catechin, green tea, non-alcoholic fatty liver disease, high-fat diet.

## Abstract

The global rise in obesity has contributed to the increasing prevalence of metabolic dysfunction-associated fatty liver disease (MAFLD, formerly known as non-alcoholic fatty liver disease, NAFLD), which is strongly linked to insulin resistance and progression to advanced liver diseases. Dietary factors are thought to influence its development and progression. We aimed to investigate the effect of green tea containing inulin and catechin (IC-GT) on high-fat diet (HFD)-induced MAFLD. Male C57BL/6 mice were assigned to five groups (10 mice/group): control (0 mg IC-GT/kg BW/d), HFD (0 mg IC-GT/kg BW/d), IC-GT-0.5X (1,328 mg IC-GT/kg BW/d), IC-GT-1X (2,645 mg IC-GT/kg BW/d), and IC-GT-2X (5,289 mg IC-GT/kg BW/d). All mice in each group were gavage-fed, received water or test samples for one week, and were then fed an HFD for 18 weeks. Blood and liver tissues were analyzed for lipid profile, enzyme activities, and histopathology. We found that IC-GT supplementation for 18 consecutive weeks significantly reduced aspartate transaminase and alanine transaminase activity as well as total cholesterol, triglyceride, low-density lipoprotein cholesterol, and high-density lipoprotein cholesterol concentrations in the blood after HFD intake, and decreased the accumulation of TC and TG in the liver. In addition, histopathological analyses showed a significant reduction in hepatic lipid droplet accumulation. Therefore, our findings suggest that long-term supplementation of IC-GT can significantly ameliorate HFD-induced MAFLD.

## Introduction

Metabolic dysfunction-associated fatty liver disease (MAFLD), formerly known as non-alcoholic fatty liver disease (NAFLD), was proposed in 2020 to more accurately reflect its pathophysiology and metabolic abnormalities 1. Although MAFLD has been recommended as the preferred term, many earlier guidelines and studies still use NAFLD. NAFLD is characterized by the presence of steatosis in more than 5% of hepatocytes and is associated with metabolic risk factors such as obesity and type 2 diabetes, after excluding excessive alcohol consumption and other chronic liver diseases 2. It represents a major cause of chronic liver disease, with an estimated global prevalence of 25.24% in the adult population 3. NAFLD encompasses a wide spectrum of conditions, ranging from simple steatosis with mild inflammation (non-alcoholic fatty liver, NAFL) to the progressive subtype non-alcoholic steatohepatitis (NASH) 4. If left untreated, NAFLD may progress to cirrhosis or hepatocellular carcinoma, and in the United States, the economic burden of managing NASH and its sequelae has been estimated to exceed USD 100 billion annually 5.

Overnutrition is a major driver of NAFLD, leading to hepatic fat accumulation and activation of inflammation, insulin resistance, lipid dysregulation, and fibrogenesis 4. Such lipid overload disrupts hepatic homeostasis, provoking oxidative stress and inflammatory signaling, which cause hepatocellular injury. These processes are reflected by elevations in alanine aminotransferase (ALT) and aspartate aminotransferase (AST), widely recognized markers of NAFLD progression 6. In parallel, insulin resistance and obesity often coincide with elevated blood glucose, triglycerides (TG), and total cholesterol (TC), further promoting steatosis and fibrosis 7. Collectively, ALT, AST, glucose, TG, TC, together with hepatic lipid accumulation, serve as key biochemical and histological markers for evaluating liver injury and metabolic dysfunction in experimental models.

Among non-pharmacological strategies, nutraceuticals and dietary supplements have attracted increasing attention. Tea contains several polyphenols, including catechins, anthocyanins, flavonoids, flavonols, and phenolic acids, with catechins accounting for approximately 60-80% of the total phenolic content 8. Catechins have been reported to regulate lipid metabolism, enhance hepatic antioxidant defenses, and reduce serum lipopolysaccharide levels 9. Carbohydrate-based dietary fibers such as inulin, lactulose, and fructooligosaccharides also provide potential benefits, as they are fermented by gut microbiota to produce metabolites including short-chain fatty acids and succinate, which contribute to lipid metabolism and appetite regulation, thereby improving obesity and fatty liver 10,11. Conversely, dysregulated fermentation of soluble dietary fibers has been shown to induce cholestasis, hepatic inflammation, and liver cancer in mice 12.

Recent clinical evidence indicates that combining catechin-rich green tea with inulin improves body composition in overweight adults 13. Mechanistically, catechins can regulate lipid metabolism and oxidative stress, whereas inulin improves glucose and lipid homeostasis through gut-liver axis modulation. These complementary actions suggest additive or synergistic potential in fatty liver disease. However, no prior study has examined this combination in an HFD-induced MAFLD model. Therefore, the present study aimed to evaluate the hepatoprotective effects of IC-GT supplementation by assessing ALT, AST, glucose, TG, TC, hepatic lipid content, and histopathology, providing mechanistic and translational insights into its potential as a nutritional strategy for MAFLD management.

## Methods

### Materials

**Figure 1:**
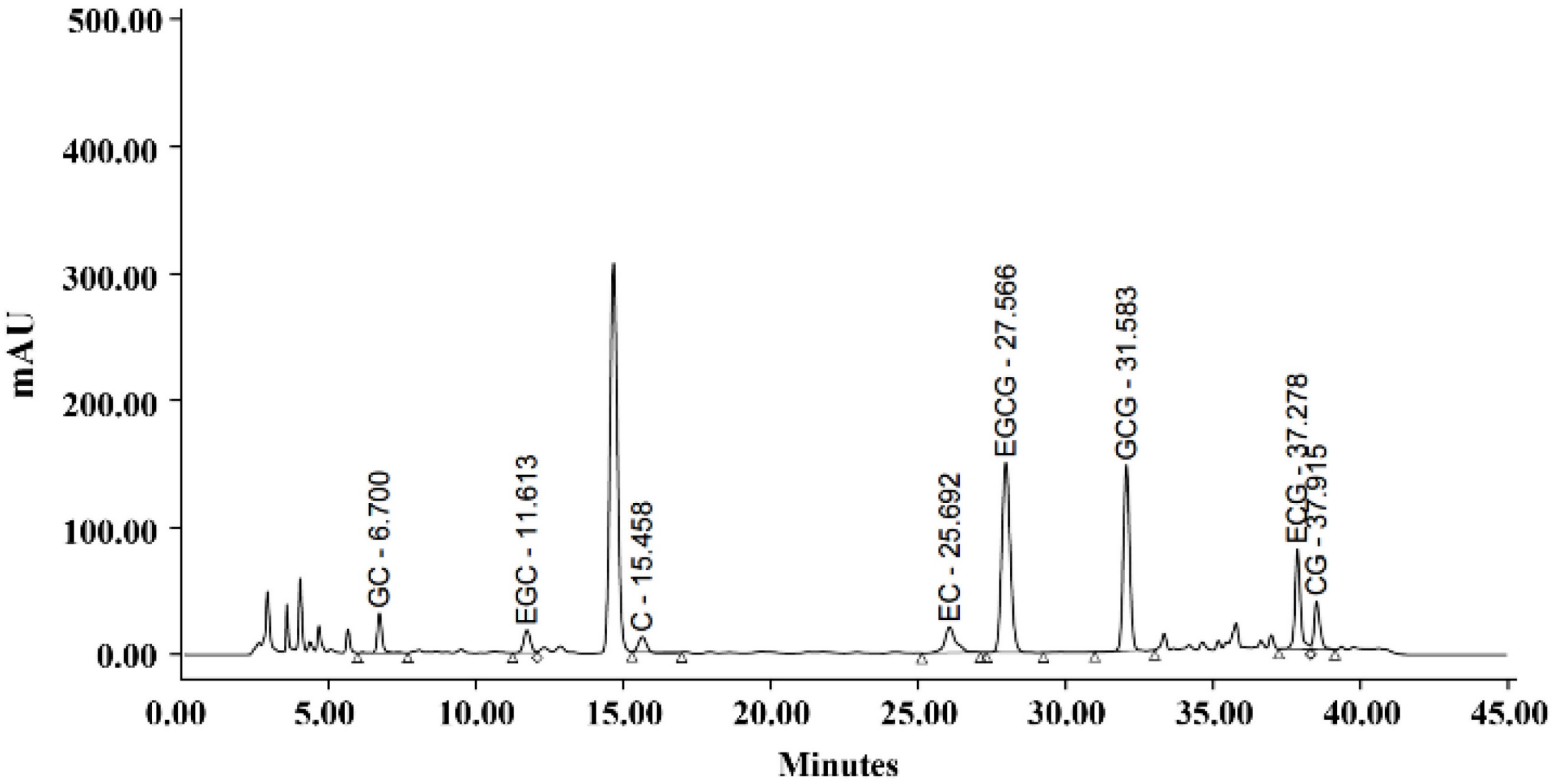
HPLC chromatogram of catechin composition.

The IC-GT supplement used in this study was a commercially available green tea beverage containing both inulin and catechins. According to the manufacturer's specifications, the product provides approximately 61.6-92.4 mg catechins per 100 mL and 1.8-2.5 g inulin per 100 mL. The catechin composition was determined using high-performance liquid chromatography, and the results are presented in Figure 1.

The recommended daily intake of IC-GT for adults is 650 ml or the equivalent 12.9 g freeze-dried powder; the dosage of IC-GT for mice was calculated based on a human with a body weight of 60 kg. The metabolic conversion based on body area in mice was 12.3, and the 1× recommended daily dose was calculated as follows: 12.9(g)/60(kg) = 0.215 × 12.3 = mouse dose 2,645 mg/kg. This formula is in agreement with US FDA guidelines. The commercial product used was a sugar-free formulation.

### Animals and treatment

The C57BL/6 (B6) mice (BioLASCO, Yi-Lan, Taiwan) weighing approximately 20- 25 g were housed in cages at 22 ± 2 °C and 65 ± 5% relative humidity with a 12-hour dark/light cycle and *ad libitum* access to food (No. 5001; PMI Nutrition International, Brentwood, MO, USA) and reverse osmosis-purified water. The experimental protocol was approved by the Animal Care and Use Committee No. 10919 for the ethical use of animals in experiments at National Taiwan Sport University. In total, 50 male eight-week-old B6 mice were randomly assigned to five groups (n = 10 per group): control (CON), HFD, HFD with half the dose of IC-GT supplement (1,328 mg/kg body weight [BW]/d, IC-GT-0.5X), HFD with a standard dose of IC-GT supplement (2,645 mg/kg BW/d, IC-GT-1X), HFD with twice the standard dose of IC-GT supplement (5,289 mg/kg BW/d, IC-GT-2X). The IC-GT supplement or vehicle (water) was administered daily via oral gavage for the entire 19-week study period. One week after the start of supplementation, all groups except the Control group were switched from a standard diet to a HFD (D12492; Research Diets Inc., New Brunswick, NJ, USA) for the remaining 18 weeks. Blood samples were collected from all mice before re-intervention, and blood and liver tissues were collected after euthanasia at the end of the experiment for lipid and histopathological analyses. A comparison of the feed ingredients is shown in Table 1.

### Measurement of biochemical parameters

At weeks 0 and 19, blood samples were collected from the submandibular (facial) vein after a 10-hour fasting period. All samples were centrifuged at 1500 × g for 15 minutes at 4 °C to separate serum. Serum levels of alanine aminotransferase (ALT), aspartate aminotransferase (AST), total cholesterol (TC), triacylglycerol (TG), and glucose were analyzed using an automated biochemical analyzer (Hitachi 7060, Hitachi, Tokyo, Japan).

### Hepatic lipid profile assay

At the end of the study, all mice were fasted for 10 hours, anesthetized with 95% CO₂, and euthanized. Blood was then collected by cardiac puncture. Subsequently, the organs were carefully removed, and the livers were excised, weighed, minced, and fixed in 10% neutral buffered formalin for histological analysis. The liver tissue was embedded in paraffin and cut into four-micrometer-thick slices for morphological and pathological evaluations. Part of the liver tissue was stored in liquid nitrogen for total cholesterol (TC) and triglyceride (TG) content analyses. A portion of the liver was analyzed using a triacylglycerol fluorometric assay kit (product number: 10010303; Cayman Chemical Co., Ann Arbor, MI, USA). Another part of the liver was homogenized in 2 ml of cold buffer (chloro-form/isopropanol/NP40 = 7:11:0.1), and the samples were centrifuged at 10,000× g for 10 min at 4 °C for analysis. After removing the supernatants, the samples were mixed with dilution buffer (50 mM sodium phosphate, pH 7.2). TC content was determined using a total cholesterol and cholesteryl ester colorimetric/fluorometric assay kit (product number: K603-100; BioVision Co., Waltham, MA, USA).

### Pathological examination of liver tissues

Liver tissues were embedded in paraffin and cut into 0.5 × 0.5 cm sections with a thickness of 4 μm for morphological and pathological evaluations. Hematoxylin and eosin (H&E) staining was performed on the sections, which were examined under a light microscope equipped with a charge-coupled device camera (BX-51; Olympus, Tokyo, Japan) by a clinical pathologist to evaluate chronic liver damage, including hepatocyte gross necrosis, fatty liver changes, and fibrosis. Steatosis level and inflammatory cell infiltration were assessed using semi-quantitative histological assessments. The grade of liver injury ranged from 0 to 4, according to the proportion of fat (fat droplets) accumulated in the liver, where 0 = 0%, 1 = <10%, 2 = 10-33%, 3 = 33-66%, and 4 = 66-100% 14.

### Statistics analysis

One-way analysis of variance (ANOVA) was used to evaluate the differences be-tween groups. A Cochran-Armitage test with SAS 9.0 software (SAS, Cary, NC, USA) was used to estimate the dose effect; p-values < 0.05 indicated significance. Dose-effect trend analysis was performed using the Cochran-Armitage test. Significance was set at *p* < 0.05.

## Results

### Effects of IC-GT on body weight gain in HFD-fed mice

**Figure 2:**
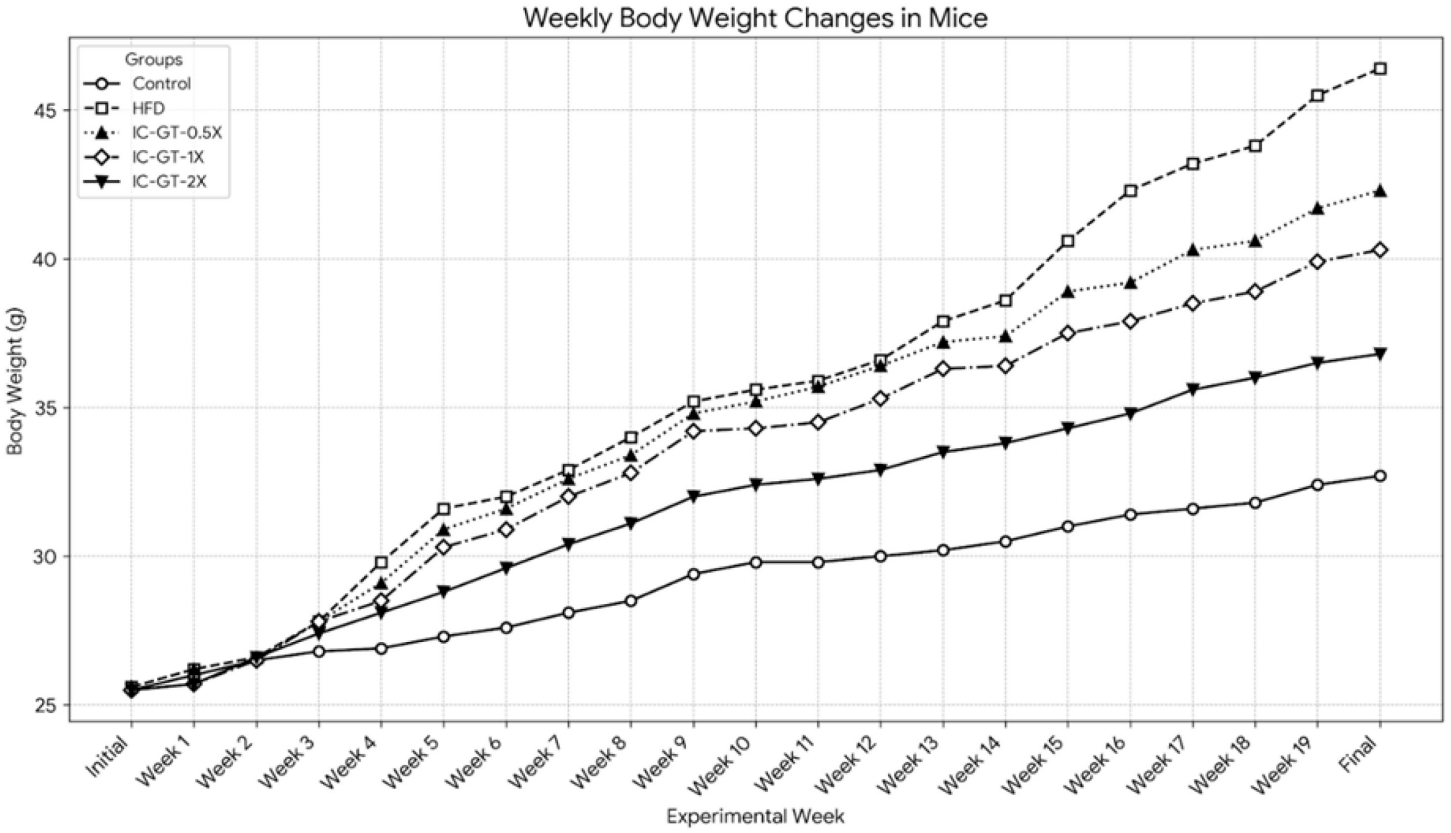
The effect of IC-GT supplementation on weekly body weight changes in mice fed a high-fat diet for 19 weeks. Values are presented as the mean body weight (g) for each group (n=10). Each line represents a different experimental group, distinguished by unique markers and line styles. The groups include: Control (standard diet), HFD (high-fat diet), and HFD supplemented with a half dose (IC-GT-0.5X), a standard dose (IC-GT-1X), or a double dose (IC-GT-2X) of the test product. HFD, high-fat diet; IC-GT, green tea containing inulin and catechins.

The weekly changes in body weight for all groups are presented in Figure 2 and Table 2. At the beginning of the study, the initial body weights were not significantly different among the five groups (*p* > 0.05). After 19 weeks of intervention, the final body weight of the High-Fat Diet (HFD) group (46.4 ± 5.0 g) was significantly higher than that of the Control group (32.7 ± 1.6 g) (*p* < 0.05). The body weight of the HFD group began to show a significant increase compared to the Control group from the fourth week of the experiment onwards.

Supplementation with IC-GT significantly attenuated the body weight gain induced by the HFD. At the end of the experiment, the final body weights of the IC-GT-0.5X (42.3 ± 2.6 g), IC-GT-1X (40.3 ± 4.2 g), and IC-GT-2X (36.8 ± 3.4 g) groups were all significantly lower than that of the HFD group (*p* < 0.05). A clear dose-dependent effect was observed, as higher IC-GT doses led to progressively greater suppression of HFD-induced weight gain, with the IC-GT-2X group showing the strongest reduction compared with all other HFD-fed groups.

### Effect of IC-GT supplementation on daily water intake, food intake, and energy intake in mice with HFD-induced liver damage

Daily food and water intake were monitored throughout the study, and the results are summarized in Table 2. HFD-fed mice consumed significantly less food by weight compared with the control group (*p* < 0.05). However, because of the higher caloric density of the HFD, their daily energy intake was significantly higher than that of controls (*p* < 0.05). IC-GT supplementation did not significantly alter food intake or energy intake compared with the HFD group.

### Effect of IC-GT supplementation on blood biochemistry in mice with HFD-induced liver damage

At week 0, there were no significant differences in baseline blood biochemical data between the groups (Table 3). At week 19, higher serum levels of aspartate transaminase (AST) (4.91-fold, *p* < 0.0001), alanine transaminase (ALT) (2.93-fold, *p* < 0.0001), glucose (2.76-fold, *p* < 0.0001), TG (2.31-fold, *p* < 0.0001), and TC (2.33-fold, *p* < 0.0001) were found in the HFD group than in the CON group. At the same time, these negative effects were attenuated in the IC-GT supplementation groups. Serum AST levels were significantly decreased by 50.89% (*p* < 0.0001), 54.20% (*p* < 0.0001), and 58.52% (*p* < 0.0001) in the IC-GT-0.5X, IC-GT-1X, and IC-GT-2X groups compared to that in the HFD group, respectively. Serum ALT levels were significantly decreased by 13.33% (*p* < 0.0001), 20.00% (*p* < 0.0001), and 31.67% (*p* < 0.0001) in the IC-GT-0.5X, IC-GT-1X, and IC-GT-2X groups compared to that in HFD groups, respectively. Serum glucose levels were significantly decreased by 14.54% (*p* < 0.0001), 16.31% (*p* < 0.0001), and 27.30% (*p* < 0.0001) in the IC-GT-0.5X, IC-GT-1X, and IC-GT-2X groups compared to that in the HFD group, respectively. Serum TG levels were significantly decreased by 29.38% (*p* < 0.0001), 29.90% (*p* < 0.0001), and 41.24% (*p* < 0.0001) in the IC-GT-0.5X, IC-GT-1X, and IC-GT-2X groups compared to that in the HFD group, respectively. Serum TC levels were significantly decreased by 11.42% (*p* = 0.0001), 17.35% (*p* < 0.0001), and 25.11% (*p* < 0.0001) in the IC-GT-0.5X, IC-GT-1X and IC-GT-2X groups compared to that in the HFD group, respectively. These findings demonstrate a clear dose-response relationship across all biochemical markers, with higher doses of IC-GT producing greater protective effects against HFD-induced metabolic disturbances (Table 3).

### Effects of IC-GT supplementation on hepatic lipid profiles in mice with HFD-induced liver damage

After HFD supplementation for 18 weeks, the TG level in the liver was significantly increased by 2.39-fold in the HFD group compared to that in the control group (p < 0.0001). Further, the TC level in the liver was 3.12-fold higher in the HFD group than in the control group (*p* < 0.0001). Protective effects were observed in the IC-GT supplementation groups. The TG levels in the liver were significantly decreased by 30.04% (*p* < 0.0001), 39.56% (*p* < 0.0001), and 56.04% (*p* < 0.0001) in the IC-GT-0.5X, IC-GT-1X, and IC-GT-2X groups compared to that in the HFD group, respectively. The TC levels in the liver were significantly decreased by 32.05% (*p* < 0.0001), 42.31% (*p* < 0.0001), and 46.15% (*p* < 0.0001) in the IC-GT-0.5X, IC-GT-1X, and IC-GT-2X groups compared to that in the HFD group, respectively. These results demonstrate a clear dose-response relationship, with progressive reductions in both hepatic TG and TC levels as the IC-GT dose increased (Table 4).

### Effects of IC-GT supplementation on liver weight changes in mice with HFD-induced liver damage

The relative weight of the liver was 1.10-fold higher in the HFD group than in the control group (*p* = 0.0289). When supplemented with twice the recommended dose of IC-GT, the relative weight of the liver was significantly reduced by 9.25% (*p* = 0.0289). However, no significant protective effects were observed in the IC-GT-0.5X and IC-GT-1X groups (Table 5).

### Liver histopathology evaluation of effect of IC-GT on mice with HFD-induced liver damage

**Figure 3:**
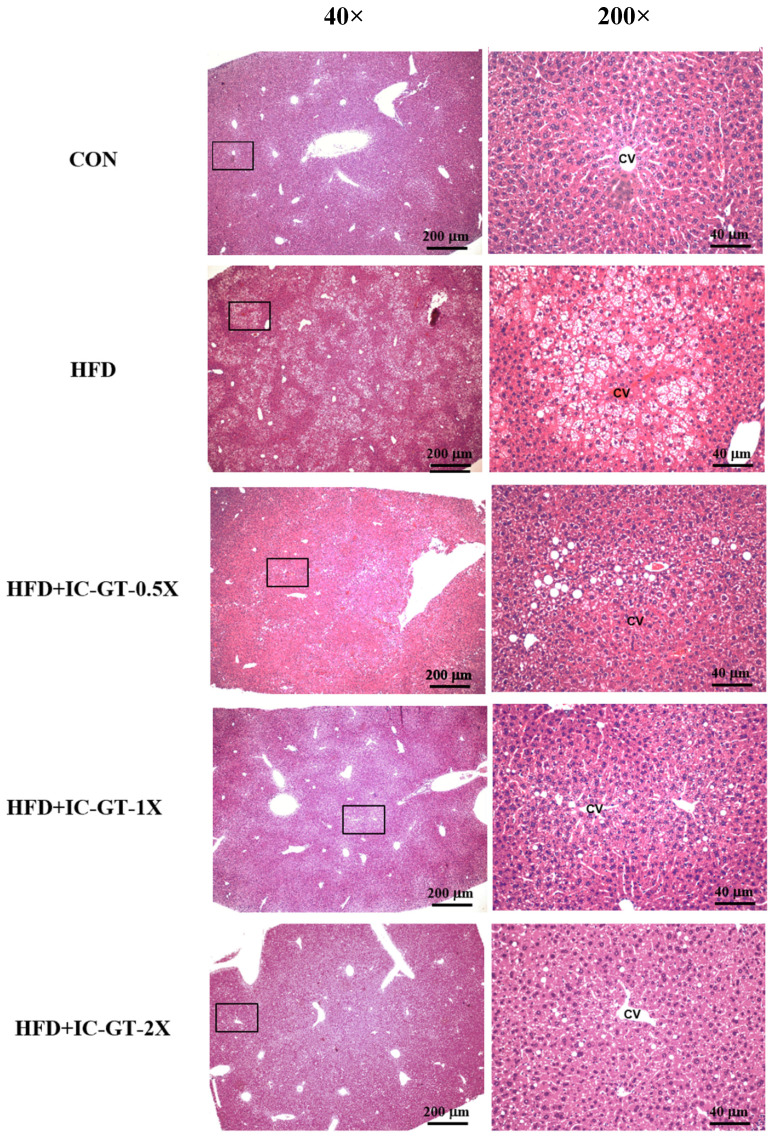
Representative hematoxylin and eosin (H&E) staining of liver tissues from the control (CON), high-fat diet (HFD), and intervention groups supplemented with green tea extract and inulin (HFD+IC-GT-0.5X, HFD+IC-GT-1X, and HFD+IC-GT-2X). Images on the left were captured at 40× magnification (scale bar = 200 μm), and those on the right at 200× magnification (scale bar = 40 μm). CV, central vein; CON, control; HFD, high-fat diet; IC-GT, catechin-rich green tea plus inulin; IC-GT-0.5X, HFD and half of the standard dose of IC-GT; IC-GT-1X, HFD and a standard dose of IC-GT; IC-GT-2X, HFD and twice the standard dose of IC-GT.

Histopathological examination revealed considerable hepatic steatosis around the central vein in the HFD group (Figure 3). In contrast to the HFD group, the IC-GT-0.5X, IC-GT-1X, and IC-GT-2X groups showed a considerable decrease in fatty liver changes; furthermore, supplementation of a higher dose was more effective in preventing lipid accumulation in the liver (Table 6).

## Discussion

The effect of IC-GT on MAFLD has not been previously investigated. Therefore, we examined the effects of different doses of IC-GT in an HFD-induced MAFLD mouse model. Our results demonstrated that a 60% HFD for 18 weeks successfully induced MAFLD, as evidenced by elevated serum TG, TC, AST, ALT, and glucose levels, along with marked hepatic lipid accumulation confirmed by histopathological analysis. Supplementation with IC-GT at various doses attenuated these adverse effects of HFD. Histopathological analysis further confirmed that IC-GT reduced hepatic lipid accumulation. In addition, the observed improvements in body weight and blood glucose may be related to enhanced lipid metabolism and improved insulin sensitivity induced by IC-GT supplementation. These biochemical improvements are particularly relevant, as elevations of ALT, AST, glucose, TG, and TC are well-established indicators of hepatocellular injury and metabolic dysfunction in NAFLD progression 6. These findings are consistent with prior reports showing that diets with 40-70% fat for 12-16 weeks are sufficient to induce obesity, steatosis, and NASH in mice 15,16. Importantly, supplementation with IC-GT prevented these pathological changes in a clear dose-dependent manner, underscoring its hepatoprotective potential.

The main finding of this study is that IC-GT-0.5X, IC-GT-1X, and IC-GT-2X supplementation effectively prevented HFD-induced MAFLD. According to the “multiple-hit” hypothesis, hepatic fat accumulation promotes lipotoxicity, mitochondrial dysfunction, oxidative stress, and inflammation, while insulin resistance, adipocyte proliferation, and gut microbiome alterations further accelerate progression 17-20. IC-GT attenuated elevations of ALT, AST, TG, TC, and glucose, suggesting both reduced hepatic lipid deposition and improved systemic metabolic regulation. These effects may reflect complementary actions: catechins enhance lipid oxidation and antioxidant defenses, while inulin improves glucose and lipid metabolism through the gut-liver axis 21-23. Notably, the reductions in ALT and AST point to direct hepatocellular protection, whereas improvements in glucose, TG, and TC indicate systemic benefits beyond weight reduction. This highlights IC-GT as a direct modulator of hepatic and metabolic homeostasis. Mechanistically, ALT and AST decreases suggest alleviation of oxidative and inflammatory stress in hepatocytes, while TG and TC reductions support improved β-oxidation and bile acid regulation. Glucose reduction further aligns with inulin's known effects on insulin sensitivity. Collectively, these biomarker changes provide mechanistic evidence that IC-GT mitigates lipotoxicity, reduces hepatocellular stress, and restores metabolic balance. The consistent dose-dependent effects across weight, biochemical, and histological findings strengthen the evidence that IC-GT exerts true biological activity rather than incidental outcomes.

Catechin, the predominant flavonoid in green tea, has been widely studied and shown to exert diverse biological activities, including antioxidant, metabolic, and cardiometabolic benefits 24-30. These observations are consistent with previous findings. A recent systematic review and meta-analysis reported that catechin supplementation can reduce body mass index, insulin resistance, and TG levels in patients with NAFLD and is generally well tolerated 31. Another meta-analysis demonstrated that green tea intake moderately lowers liver enzyme levels in patients with NAFLD 32. Several mechanisms have been proposed to explain these effects. Green tea supplementation has been shown to enhance hepatic acyl-CoA oxidase and medium-chain acyl-CoA dehydrogenase mRNA expression, increase β-oxidation activity, reduce hepatic COX-2 and prostaglandin E2 levels, and protect against NFκB activation in NAFLD animal models 33-36. One study showed that epigallocatechin supplementation for 14 weeks not only prevented hepatic fat accumulation, abnormal liver function, and elevated inflammatory cytokines in HFD-fed mice but also improved intestinal mucosal immunity 37. Another study demonstrated that catechins, particularly epigallocatechin and epicatechin, activate peroxisome proliferator-activated receptor alpha, inhibit lipogenic pathways, and induce β-oxidation in HepG2 cells through indirect mechanisms 38.

Inulin exerts beneficial effects by modulating the gut microbiome 39 and has been shown to improve serum lipid levels, fasting blood glucose, and insulin resistance 40-43. One study reported that inulin suppresses the lipopolysaccharide-Toll-like receptor 4-macrophage-NF-κB-NLRP3 inflammatory pathway in mice 44. Another investigation demonstrated that inulin enhances bile acid excretion by restoring farnesoid X receptor activity and increasing *de novo* hepatic bile acid synthesis 45. In our study, inulin was incorporated into catechin-rich green tea because of its potential hepatoprotective effects against MAFLD. The tested doses were well tolerated, and no adverse events were observed. To the best of our knowledge, this is the first study to evaluate the effects of combined catechin and inulin supplementaion on MAFLD. The dosages of catechin and inulin used in this study fall within the ranges commonly reported in preclinical studies investigating their metabolic effects. For example, dietary inulin at 5-10% of the diet has been shown to reduce hepatic lipid deposition and improve insulin sensitivity in mice, largely through gut microbiota-derived short-chain fatty acids and bile acid regulation 23. Similarly, catechin or green tea extract supplementation at comparable doses has been demonstrated to reduce hepatic steatosis and improve serum transaminases in NAFLD models, consistent with its ability to modulate lipid metabolism and attenuate oxidative and inflammatory signaling 21-22. These complementary mechanisms of inulin and catechins may underlie the dose-dependent hepatoprotective effects observed in our study.

Our findings provide the first evidence that combined catechin-enriched green tea and inulin supplementation protects against HFD-induced MAFLD. Nevertheless, several limitations must be acknowledged. First, clinical trials are needed to confirm the translational efficacy of IC-GT. Second, because catechin-only or inulin-only groups were not included, the relative contribution of each cannot be determined. Third, although a dose-response trend was demonstrated after 18 weeks, the optimal dose and intervention period remain to be established. Finally, molecular analyses were not performed; future studies should investigate gene and protein expression related to lipid metabolism, oxidative stress, and inflammatory pathways to elucidate IC-GT's mechanisms 46.

## Conclusions

This study demonstrated that 18 weeks of IC-GT supplementation effectively mitigated the adverse effects of an HFD in mice. The improvements were evident in reduced serum transaminases, improved lipid profiles, and decreased hepatic lipid accumulation, which together support a protective effect of IC-GT against diet-induced metabolic dysfunction. Considering the well-documented roles of catechins in regulating lipid metabolism and the reported ability of inulin to improve glucose and lipid homeostasis, the hepatoprotective effects observed in this study may reflect complementary actions of these components. Overall, our findings provide experimental evidence that the combined supplementation of catechin-enriched green tea and inulin provides a basis for considering it as a potential nutritional approach for mitigating MAFLD progression.

## Figures and Tables

**Table 1:** Calories and composition of three major nutrients in standard feed and high-fat feed.

Ingredient	CON group 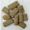	HFD group 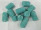
Protein (kcal%)	28.5	20
Lipid (kcal%)	13.4	60
Carbohydrate (kcal%)	58.1	20
Total energy (kcal/g)	3.36	5.24

CON, control; HFD, high-fat diet.

**Table 2:** Effect of IC-GT supplementation on body weight, daily water intake, food intake, and energy intake in mice with HFD-induced liver damage.

Characteristics	CON	HFD	IC-GT		
			0.5X	1X	2X
Initial BW (g)	25.5±0.5^a^	25.6±0.6^a^	25.6±1.4^a^	25.5±0.7^a^	25.5±0.6^a^
1st wk BW	26.0±0.6^a^	26.2±0.6^a^	26.2±1.0^a^	25.7±0.6^a^	25.7±0.5^a^
2nd wk BW	26.5±0.9^a^	26.6±0.6^a^	26.6±1.1^a^	26.5±0.8^a^	26.6±1.0^a^
3rd wk BW	26.8±1.3^a^	27.8±0.8^a^	27.8±1.2^a^	27.8±1.1^a^	27.4±1.4^a^
4th wk BW	26.9±1.3^a^	29.8±1.1^c^	29.1±1.8^bc^	28.5±1.4^b^	28.1±1.6^ab^
5th wk BW	27.3±1.6^a^	31.6±1.2^c^	30.9±2.2^c^	30.3±1.8^bc^	28.8±1.5^b^
6th wk BW	27.6±1.6^a^	32.0±1.3^c^	31.6±2.2^c^	30.9±1.9^bc^	29.6±1.5^c^
7th wk BW	28.1±1.8^a^	32.9±1.6^c^	32.6±2.6^c^	32.0±2.3^bc^	30.4±1.6^b^
8th wk BW	28.5±1.9^a^	34.0±1.9^c^	33.4±2.6^c^	32.8±2.3^bc^	31.1±1.7^b^
9th wk BW	29.4±2.0^a^	35.2±2.4^c^	34.8±2.5^c^	34.2±2.4^c^	32.0±1.7^b^
10th wk BW	29.8±1.8^a^	35.6±2.5^c^	35.2±2.7^c^	34.3±2.4^bc^	32.4±1.7^b^
11th wk BW	29.8±1.8^a^	35.9±2.5^c^	35.7±2.7^c^	34.5±2.5^bc^	32.6±1.8^b^
12th wk BW	30.0±1.8^a^	36.6±2.7^c^	36.4±2.7^c^	35.3±2.7^c^	32.9±1.8^b^
13th wk BW	30.2±1.8^a^	37.9±2.8^c^	37.2±2.6^c^	36.3±2.9^c^	33.5±2.2^b^
14th wk BW	30.5±1.9^a^	38.6±3.1^c^	37.4±2.8^c^	36.4±3.0^c^	33.8±2.4^b^
15th wk BW	31.0±1.8^a^	40.6±3.4^d^	38.9±2.5^cd^	37.5±3.5^c^	34.3±2.7^b^
16th wk BW	31.4±1.7^a^	42.3±3.8^d^	39.2±2.5^c^	37.9±3.4^c^	34.8±2.8^b^
17th wk BW	31.6±1.7^a^	43.2±4.1^d^	40.3±2.7^c^	38.5±3.6^c^	35.6±3.4^b^
18th wk BW	31.8±1.7^a^	43.8±4.4^d^	40.6±2.5^c^	38.9±3.8^bc^	36.0±3.4^b^
19th wk BW	32.4±1.6^a^	45.5±4.8^d^	41.7±2.6^c^	39.9±4.0^c^	36.5±3.4^b^
Final BW	32.7±1.6^a^	46.4±5^d^	42.3±2.6^c^	40.3±4.2^c^	36.8±3.4^b^
Water intake (mL/mouse/day)	4.9±0.6^b^	3.0±0.6^a^	3.1±0.5^a^	3.0±0.5^a^	3.1±0.5^a^
Diet (g/mouse/day)	3.8±0.5^a^	2.7±0.7^b^	2.7±0.6^b^	2.8±0.7^b^	2.7±0.6^b^
Diet (kcal/mouse/day)	12.6±1.7^a^	14.1±3.4^b^	14.2±3.2^b^	14.6±3.4^b^	14.1±3.3^b^

Data are presented as the mean ± standard deviation for 10 mice in each group. Different letters (a, b, c, and c) indicate significant differences at *p* < 0.05, according to a one-way analysis of variance. HFD, high-fat diet; IC-GT, catechin-rich green tea plus inulin; IC-GT-0.5X, HFD with half of the standard dose of IC-GT; IC-GT-1X, HFD with a standard dose of IC-GT; IC-GT-2X, HFD with twice the standard dose of IC-GT.

**Table 3:** Effect of IC-GT supplementation on blood biochemistry in mice with HFD-induced liver damage.

Parameters	Week 0					Week 19				
	CON	HFD	IC-GT			CON	HFD	IC-GT		
			0.5X	1X	2X			0.5X	1X	2X
AST (U/L)	72±6^ a^	71±6^ a^	72±5^ a^	71±7^ a^	71±4^ a^	80±3^a^	393±48^d^	193±10^c^	180±25^bc^	163±17^c^
ALT (U/L)	33±3^ a^	34±2^ a^	34±3^ a^	32±5^ a^	33±4^ a^	41±3^a^	120±8^e^	104±8^d^	96±6^c^	82±4^b^
Glucose (mg/dL)	96±6^ a^	96±7^ a^	97±4^ a^	94±6^ a^	97±6^ a^	102±6^a^	282±9^d^	241±5^c^	236±7^c^	205±6^b^
TG (mg/dL)	83±4^ a^	83±5^ a^	84±7^ a^	84±6^ a^	83±6^ a^	84±7^a^	194±11^d^	137±7^c^	136±5^c^	114±8^b^
TC (mg/dL)	92±3^ a^	92±5^ a^	91±7^ a^	94±3^ a^	93±3^ a^	94±3^a^	219±7^e^	194±8^d^	181±5^c^	164±8^b^

Data are presented as the mean ± standard deviation for 10 mice in each group. Different letters (a, b, c, d, and e) indicate significant differences at *p* < 0.05, according to a one-way analysis of variance. HFD, high-fat diet; IC-GT, catechin-rich green tea plus inulin; IC-GT-0.5X, HFD with half of the standard dose of IC-GT; IC-GT-1X, HFD with a standard dose of IC-GT; IC-GT-2X, HFD with twice the standard dose of IC-GT; AST, aspartate transaminase; ALT, alanine transaminase; TG, triglyceride; TC, total cholesterol.

**Table 4:** Hepatic lipid profile after IC-GT supplementation at week 19.

Hepatic lipid	Control	HFD	IC-GT		
			0.5X	1X	2X
TG (mg/g liver)	11.4 ± 1.11^a^	27.3 ± 2.36^d^	19.1 ± 1.54^c^	16.5 ± 2.76^b^	12.0 ± 1.59^a^
TC (mg/g liver)	2.49 ± 0.27^a^	7.84 ± 0.99^d^	5.29 ± 0.24^c^	4.53 ± 0.38^b^	4.23 ± 0.24^b^

Data are presented as the mean ± standard deviation for 10 mice in each group. Different letters (a, b, c, d, and e) indicate significant differences at *p* < 0.05, according to a one-way analysis of variance. HFD, high-fat diet; IC-GT, catechin-rich green tea plus inulin; IC-GT-0.5X, HFD with half of the standard dose of IC-GT; IC-GT-1X, HFD and a standard dose of IC-GT; IC-GT-2X, HFD with twice the standard dose of IC-GT; AST, aspartate transaminase; ALT, alanine transaminase; TG, triglyceride; TC, total cholesterol.

**Table 5:** Liver/body weight proportion after IC-GT supplementation at week 19.

Characteristic	Control	HFD	IC-GT		
			0.5X	1X	2X
Liver/BW%	3.14 ± 0.15^a^	3.46 ± 0.17^b^	3.44 ± 0.48^b^	3.30 ± 0.41^ab^	3.14 ± 0.26^a^

Data are presented as the mean ± standard deviation for 10 mice in each group. Different letters (a, b) indicate significant differences at *p* < 0.05 according to the one-way analysis of variance. BW, body weight; HFD, high-fat diet; IC-GT, catechin-rich green tea plus inulin; IC-GT-0.5X, HFD and half of the standard dose of IC-GT; IC-GT-1X, HFD and standard dose of IC-GT; IC-GT-2X, HFD and twice the standard dose of IC-GT.

**Table 6:** Effects of IC-GT supplementation on histopathology of liver tissue at week 19.

Hepatic lipid	Control	HFD	IC-GT		
			0.5X	1X	2X
Hepatic histopathological analysis scores	0 ± 0^a^	2.90 ± 0.32^c^	1.60 ± 0.84^b^	0.40 ± 0.52^a^	0.10 ± 0.32^a^

Data are presented as the mean ± standard deviation for 10 mice in each group. Different letters (a, b, and c) indicate significant differences at p < 0.05 according to a one-way analysis of variance. HFD, high-fat diet; IC-GT-0.5X, HFD and half of the standard dose of IC-GT; IC-GT-1X, HFD and a standard dose of IC-GT; IC-GT-2X, HFD and twice the standard dose of IC-GT. Fatty liver score defined the severity of fatty liver changes; 4: extremely severe, steatosis in >66% liver cells; 3: severe, steatosis in 33-66% liver cells; 2: moderate, steatosis in 10-33% liver cells; 1: mild, steatosis in <10% liver cells; 0: normal, steatosis in 0% liver cells.
